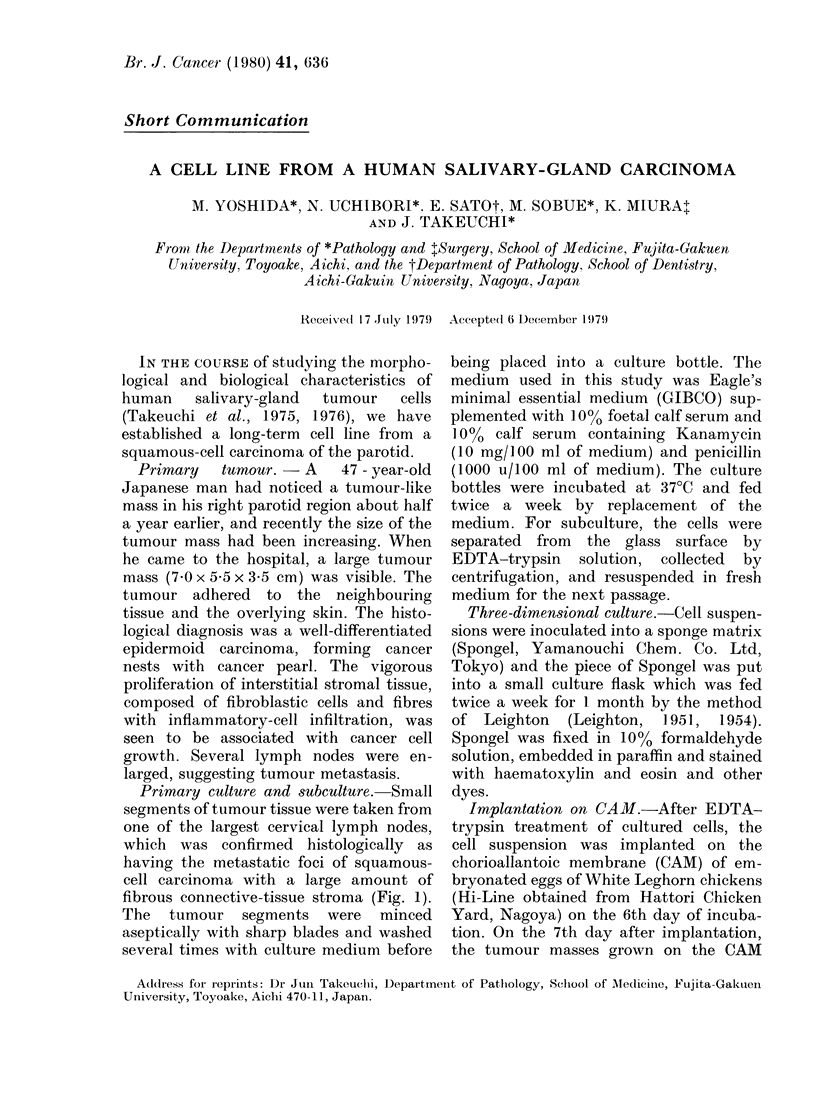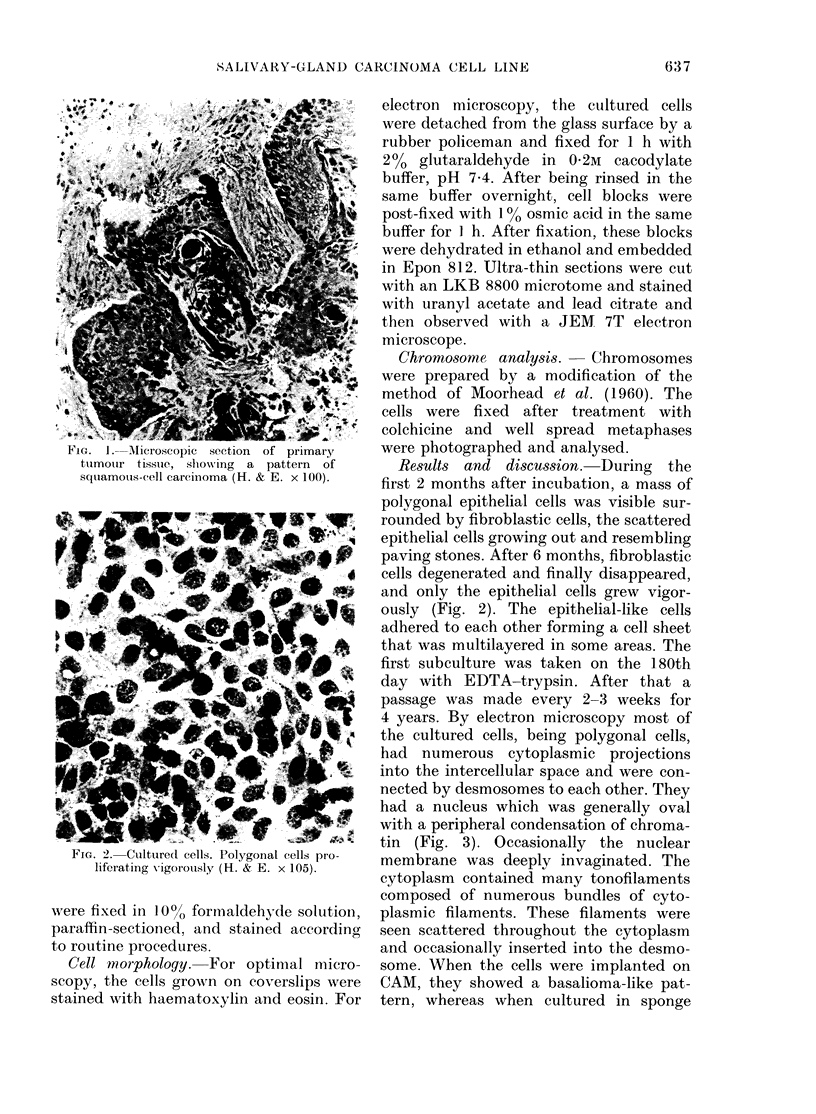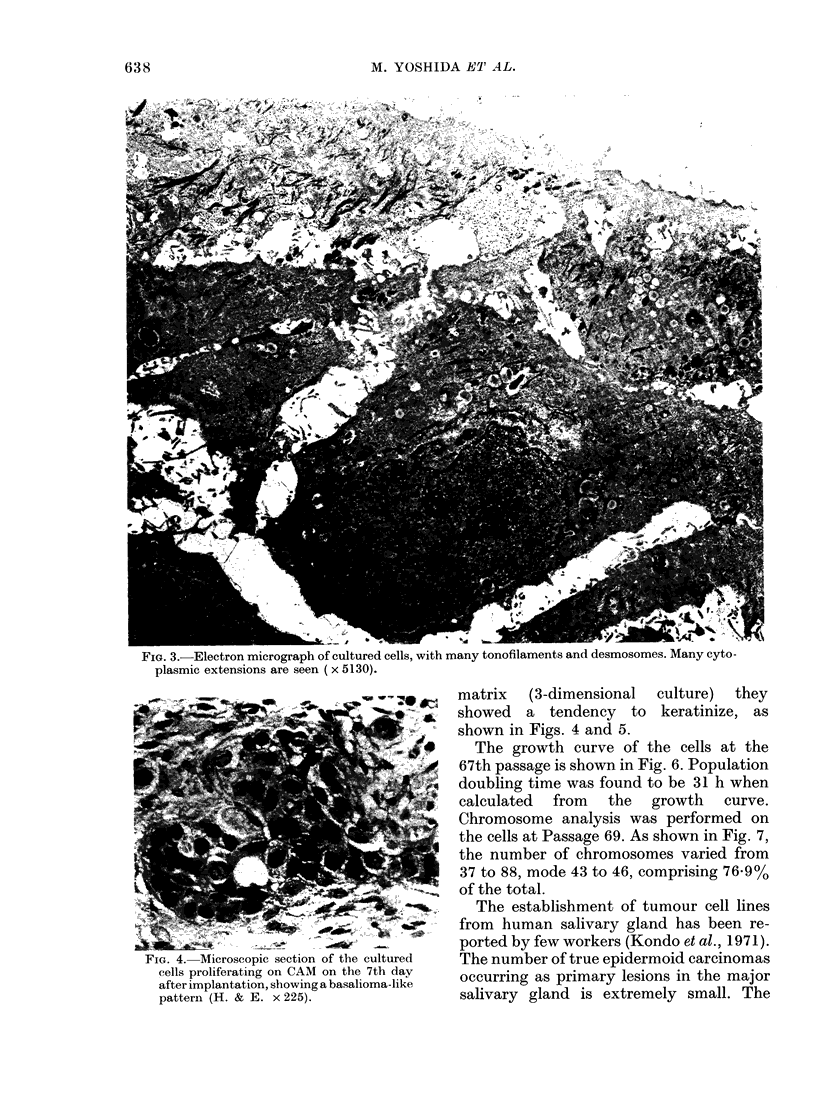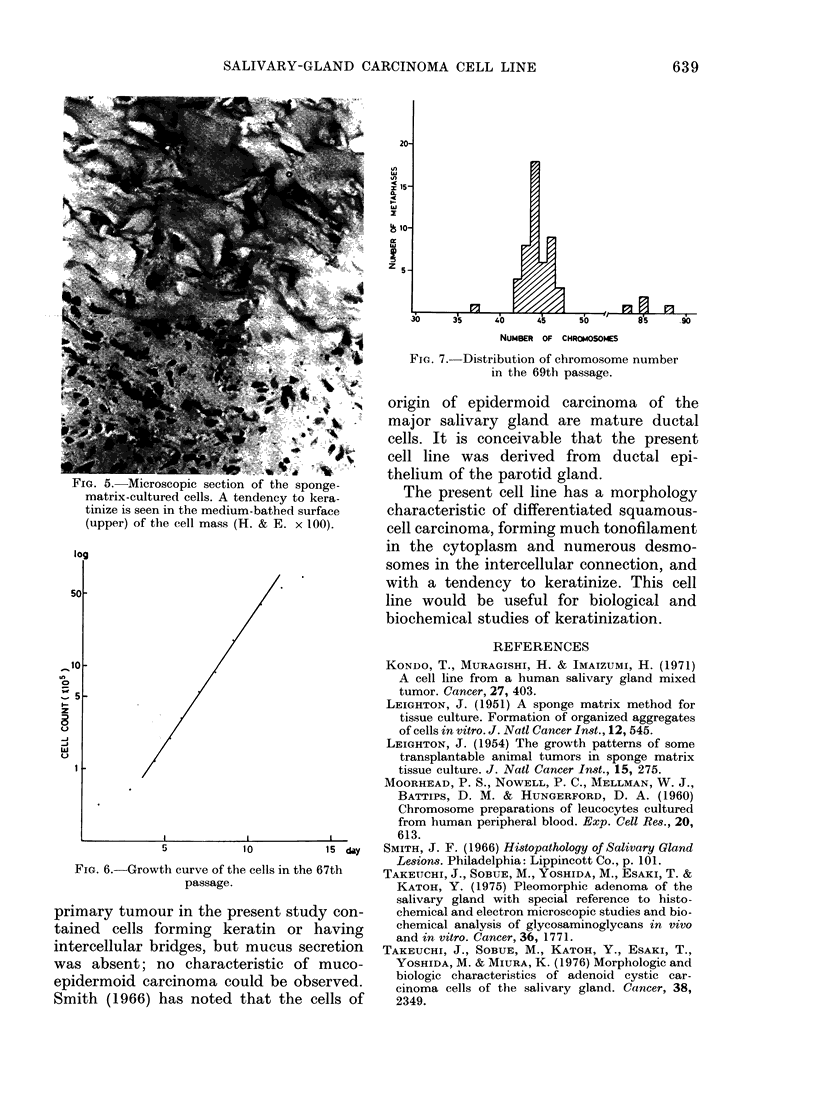# A cell line from a human salivary-gland carcinoma.

**DOI:** 10.1038/bjc.1980.107

**Published:** 1980-04

**Authors:** M. Yoshida, N. Uchibori, E. Sato, M. Sobue, K. Miura, J. Takeuchi

## Abstract

**Images:**


*Br. J. Cancer* (1980) **41**, 636

***Short Communication***

### A CELL LINE FROM A HUMAN SALIVARY-GLAND CARCINOMA

M. YOSHIDA*, N. UCHIBORI*, E. SATO†, M. SOBUE*, K. MIURA‡ AND J. TAKEUCHI*

*From the Departments of *Pathology and ‡Surgery, School of Medicine, Fujita-Gakuen University, Toyoake, Aichi, and the †Department of Pathology, School of Dentistry, Aichi-Gakuin University, Nagoya, Japan*

Received 17 July 1979 Accepted 6 December 1979

IN THE COURSE of studying the morphological and biological characteristics of human salivary-gland tumour cells (Takeuchi *et al.*, 1975, 1976), we have established a long-term cell line from a squamous-cell carcinoma of the parotid.

*Primary tumour.* — A 47-year-old Japanese man had noticed a tumour-like mass in his right parotid region about half a year earlier, and recently the size of the tumour mass had been increasing. When he came to the hospital, a large tumour mass (7·0 × 5·5 × 3·5 cm) was visible. The tumour adhered to the neighbouring tissue and the overlying skin. The histological diagnosis was a well-differentiated epidermoid carcinoma, forming cancer nests with cancer pearl. The vigorous proliferation of interstitial stromal tissue, composed of fibroblastic cells and fibres with inflammatory-cell infiltration, was seen to be associated with cancer cell growth. Several lymph nodes were enlarged, suggesting tumour metastasis.

*Primary culture and subculture.*—Small segments of tumour tissue were taken from one of the largest cervical lymph nodes, which was confirmed histologically as having the metastatic foci of squamous-cell carcinoma with a large amount of fibrous connective-tissue stroma (Fig. 1). The tumour segments were minced aseptically with sharp blades and washed several times with culture medium before being placed into a culture bottle. The medium used in this study was Eagle's minimal essential medium (GIBCO) supplemented with 10% foetal calf serum and 10% calf serum containing Kanamycin (10 mg/100 ml of medium) and penicillin (1000 u/100 ml of medium). The culture bottles were incubated at 37°C and fed twice a week by replacement of the medium. For subculture, the cells were separated from the glass surface by EDTA–trypsin solution, collected by centrifugation, and resuspended in fresh medium for the next passage.

*Three-dimensional culture.*—Cell suspensions were inoculated into a sponge matrix (Spongel, Yamanouchi Chem. Co. Ltd, Tokyo) and the piece of Spongel was put into a small culture flask which was fed twice a week for 1 month by the method of Leighton (Leighton, 1951, 1954). Spongel was fixed in 10% formaldehyde solution, embedded in paraffin and stained with haematoxylin and eosin and other dyes.

*Implantation on CAM.*—After EDTA–trypsin treatment of cultured cells, the cell suspension was implanted on the chorioallantoic membrane (CAM) of embryonated eggs of White Leghorn chickens (Hi-Line obtained from Hattori Chicken Yard, Nagoya) on the 6th day of incubation. On the 7th day after implantation, the tumour masses grown on the CAM

Address for reprints: Dr Jun Takeuchi, Department of Pathology, School of Medicine, Fujita-Gakuen University, Toyoake, Aichi 470-11, Japan.

FIG. 1.—Microscopic section of primary tumour tissue, showing a pattern of squamous-cell carcinoma (H. & E. ×100).

FIG. 2.—Cultured cells. Polygonal cells proliferating vigorously (H. & E. ×105).

were fixed in 10% formaldehyde solution, paraffin-sectioned, and stained according to routine procedures.

*Cell morphology.*—For optimal microscopy, the cells grown on coverslips were stained with haematoxylin and eosin. For electron microscopy, the cultured cells were detached from the glass surface by a rubber policeman and fixed for 1 h with 2% glutaraldehyde in 0·2M cacodylate buffer, pH 7·4. After being rinsed in the same buffer overnight, cell blocks were post-fixed with 1% osmic acid in the same buffer for 1 h. After fixation, these blocks were dehydrated in ethanol and embedded in Epon 812. Ultra-thin sections were cut with an LKB 8800 microtome and stained with uranyl acetate and lead citrate and then observed with a JEM 7T electron microscope.

*Chromosome analysis.* — Chromosomes were prepared by a modification of the method of Moorhead *et al.* (1960). The cells were fixed after treatment with colchicine and well spread metaphases were photographed and analysed.

*Results and discussion.*—During the first 2 months after incubation, a mass of polygonal epithelial cells was visible surrounded by fibroblastic cells, the scattered epithelial cells growing out and resembling paving stones. After 6 months, fibroblastic cells degenerated and finally disappeared, and only the epithelial cells grew vigorously (Fig. 2). The epithelial-like cells adhered to each other forming a cell sheet that was multilayered in some areas. The first subculture was taken on the 180th day with EDTA–trypsin. After that a passage was made every 2–3 weeks for 4 years. By electron microscopy most of the cultured cells, being polygonal cells, had numerous cytoplasmic projections into the intercellular space and were connected by desmosomes to each other. They had a nucleus which was generally oval with a peripheral condensation of chromatin (Fig. 3). Occasionally the nuclear membrane was deeply invaginated. The cytoplasm contained many tonofilaments composed of numerous bundles of cytoplasmic filaments. These filaments were seen scattered throughout the cytoplasm and occasionally inserted into the desmosome. When the cells were implanted on CAM, they showed a basalioma-like pattern, whereas when cultured in sponge

FIG. 3.—Electron micrograph of cultured cells, with many tonofilaments and desmosomes. Many cytoplasmic extensions are seen ( × 5130).

FIG. 4.—Microscopic section of the cultured cells proliferating on CAM on the 7th day after implantation, showing a basalioma-like pattern (H. & E. × 225).

matrix (3-dimensional culture) they showed a tendency to keratinize, as shown in Figs. 4 and 5.

The growth curve of the cells at the 67th passage is shown in Fig. 6. Population doubling time was found to be 31 h when calculated from the growth curve. Chromosome analysis was performed on the cells at Passage 69. As shown in Fig. 7, the number of chromosomes varied from 37 to 88, mode 43 to 46, comprising 76·9% of the total.

The establishment of tumour cell lines from human salivary gland has been reported by few workers (Kondo *et al.*, 1971). The number of true epidermoid carcinomas occurring as primary lesions in the major salivary gland is extremely small. The

FIG. 5.—Microscopic section of the sponge-matrix-cultured cells. A tendency to keratinize is seen in the medium-bathed surface (upper) of the cell mass (H. & E. × 100).

FIG. 6.—Growth curve of the cells in the 67th passage.

primary tumour in the present study contained cells forming keratin or having intercellular bridges, but mucus secretion was absent; no characteristic of mucoepidermoid carcinoma could be observed. Smith (1966) has noted that the cells of

FIG. 7.—Distribution of chromosome number in the 69th passage.

origin of epidermoid carcinoma of the major salivary gland are mature ductal cells. It is conceivable that the present cell line was derived from ductal epithelium of the parotid gland.

The present cell line has a morphology characteristic of differentiated squamous-cell carcinoma, forming much tonofilament in the cytoplasm and numerous desmosomes in the intercellular connection, and with a tendency to keratinize. This cell line would be useful for biological and biochemical studies of keratinization.

### REFERENCES

KONDO, T., MURAGISHI, H. & IMAIZUMI, H. (1971) A cell line from a human salivary gland mixed tumor. *Cancer*, **27**, 403.

LEIGHTON, J. (1951) A sponge matrix method for tissue culture. Formation of organized aggregates of cells *in vitro*. *J. Natl Cancer Inst.*, **12**, 545.

LEIGHTON, J. (1954) The growth patterns of some transplantable animal tumors in sponge matrix tissue culture. *J. Natl Cancer Inst.*, **15**, 275.

MOORHEAD, P. S., NOWELL, P. C., MELLMAN, W. J., BATTIPS, D. M. & HUNGERFORD, D. A. (1960) Chromosome preparations of leucocytes cultured from human peripheral blood. *Exp. Cell Res.*, **20**, 613.

SMITH, J. F. (1966) *Histopathology of Salivary Gland Lesions*. Philadelphia: Lippincott Co., p. 101.

TAKEUCHI, J., SOBUE, M., YOSHIDA, M., ESAKI, T. & KATOH, Y. (1975) Pleomorphic adenoma of the salivary gland with special reference to histochemical and electron microscopic studies and biochemical analysis of glycosaminoglycans *in vivo* and *in vitro*. *Cancer*, **36**, 1771.

TAKEUCHI, J., SOBUE, M., KATOH, Y., ESAKI, T., YOSHIDA, M. & MIURA, K. (1976) Morphologic and biologic characteristics of adenoid cystic carcinoma cells of the salivary gland. *Cancer*, **38**, 2349.